# Human genes with CpG island promoters have a distinct transcription-associated chromatin organization

**DOI:** 10.1186/gb-2012-13-11-r110

**Published:** 2012-11-27

**Authors:** Tanya Vavouri, Ben Lehner

**Affiliations:** 1 Institute of Predictive and Personalized Medicine of Cancer (IMPPC), Badalona, Barcelona 08916, Spain; 2 EMBL-CRG Systems Biology Unit and ICREA, Centre for Genomic Regulation and UPF, Barcelona 08003, Spain

## Abstract

**Background:**

More than 50% of human genes initiate transcription from CpG dinucleotide-rich regions referred to as CpG islands. These genes show differences in their patterns of transcription initiation, and have been reported to have higher levels of some activation-associated chromatin modifications.

**Results:**

Here we report that genes with CpG island promoters have a characteristic transcription-associated chromatin organization. This signature includes high levels of the transcription elongation-associated histone modifications H4K20me1, H2BK5me1 and H3K79me1/2/3 in the 5' end of the gene, depletion of the activation marks H2AK5ac, H3K14ac and H3K23ac immediately downstream of the transcription start site (TSS), and characteristic epigenetic asymmetries around the TSS. The chromosome organization factor CTCF may be bound upstream of RNA polymerase in most active CpG island promoters, and an unstable nucleosome at the TSS may be specifically marked by H4K20me3, the first example of such a modification. H3K36 monomethylation is only detected as enriched in the bodies of active genes that have CpG island promoters. Finally, as expression levels increase, peak modification levels of the histone methylations H3K9me1, H3K4me1, H3K4me2 and H3K27me1 shift further away from the TSS into the gene body.

**Conclusions:**

These results suggest that active genes with CpG island promoters have a distinct step-like series of modified nucleosomes after the TSS. The identity, positioning, shape and relative ordering of transcription-associated histone modifications differ between genes with and without CpG island promoters. This supports a model where chromatin organization reflects not only transcription activity but also the type of promoter in which transcription initiates.

## Background

More than half of human genes initiate transcription from regions of the genome with an elevated content of CpG dinucleotides and G+C base pairs referred to as 'CpG islands' [1,2]. In contrast to the rest of the genome, where CpG dinucleotides are heavily methylated and so rapidly lost through deamination, CpG sites within promoter CpG islands are normally free from DNA methylation and do not have an elevated mutation rate [3-7]. Genes with promoters containing CpG islands (henceforth CpG promoter genes) encode housekeeping genes expressed in all cell types [8-11] but also include a substantial number of master developmental regulators such as *HOX *genes [9,12]. In contrast, non-CpG promoter genes tend to have more restricted expression patterns and to be expressed later in development during tissue differentiation.

Several lines of evidence suggest that the process of transcription initiation differs in CpG and non-CpG promoters. Systematic identification of the 5' ends of mammalian transcripts revealed that transcription tends to initiate from a broad region in CpG promoters but in a sharp peak in non-CpG promoters [13]. CpG promoters also more frequently initiate transcription in both the sense and antisense direction, and produce unstable non-coding RNAs even in the absence of full-length mRNA production [13-16]. Further, RNA polymerase II may be constitutively recruited to CpG promoters [14,17,18], with polymerase release being an important point of regulation [14,19]. CpG promoters are less likely to contain a TATA-box [13], and contain fewer specifically located transcription factor binding sites [20].

In addition to transcription, chromatin organization has also been reported to differ between CpG and non-CpG promoters. CpG and GC-rich DNA is preferentially bound by CXXC domain proteins that can recruit chromatin-modifying activities, including Cfp1 [21], a subunit of an H3K4me3 methyltransferase complex [22], and KDM2A, a H3K36me2 demethylase [23]. Consistent with this, unmethylated CpG promoters have higher levels of H3K4me3, a histone modification associated with transcription initiation [24-27]. However, CpG promoters also have higher levels of other modifications associated with transcription activation, such as the histone H3 lysine 4 methylations H3K4me1 and H3K4me2, and the histone variant H2A.Z [26,27]. Moreover, it has been reported that GC-rich sequences can recruit the polycomb repressive complex 2 [28]. CpG promoters have also been reported to contain a more pronounced nucleosome-depleted region upstream of the start site, despite the fact that nucleosomes have a high intrinsic affinity for G+C and CpG rich DNA [29]. This distinction between nucleosome-depleted CpG promoters and nucleosome occupied non-CpG promoters is reminiscent of the distinction between two major classes of promoter in budding yeast [30,31]. Finally, in efforts to use chromatin modifications to predict the locations of core promoters or gene expression levels, different modifications have sometimes been reported as most useful for genes with and without CpG islands [32,33]. For example, in the models developed by Karlic *et al. *[33], H4K20me1 and H3K27ac were most frequently employed to predict the expression levels of genes with CpG island promoters, whereas H3K4me3 and H3K79me1 were the modifications most frequently used in models to predict the expression levels of non-CpG island genes.

Chromatin-modifying enzymes can be recruited by elongating polymerase complexes, by sequence-specific DNA-binding proteins, and by non-coding RNAs [34]. We hypothesized, therefore, that, beyond the distinctions described above, promoter type could be quite a general influence on the chromatin organization of a gene, including distally, away from the start site. We show here that this is indeed the case, and that genes with CpG island promoters show characteristic transcription-coupled changes in chromatin organization not seen in other genes. In particular, CpG promoter genes show a distinct set of transcription-linked epigenetic transitions within the 5' end of their gene bodies. They also have a different chromatin organization within the promoter region, including a histone modification specifically detected at the initiation site. Our analyses highlight complex differences in the chromatin of human genes with and without CpG islands in their promoters, and are consistent with a model in which there are at least two characteristic ways in which the chromatin of a human gene changes from repression to activation, depending upon the type of promoter in which transcription initiates.

## Results

### Chromatin profile comparison of expression-matched genes with and without CpG islands in their promoters

**Figure 1:**
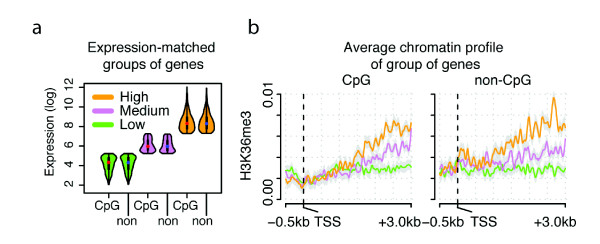
**Chromatin profile comparison of expression-matched genes with and without CpG islands in their promoters**. **(a) **Expression levels of 1,500 expression-matched CpG and non-CpG promoter genes classified as having low, medium and high expression levels (500 genes of each type in each category). **(b) **Average H3K36me3 profile between 500 bp upstream and 3 kb downstream of the transcription start site (TSS) of genes with or without a CpG promoter and with low, medium or high expression levels (color coded as in panel (a). Lines represent the loess-smoothed average read density shifted by 75 bp to indicate the position of the nucleosome dyad and the grey shading indicates the area within two standard errors of the predicted loess line.

To test whether human genes with CpG island promoters have a distinct chromatin organization, we analyzed the levels of histone modifications and other epigenetic modifications in 1,500 CpG and 1,500 non-CpG promoter genes with precisely matched expression levels (Materials and methods; Figure 1a; Additional files 1 and 2). Transcription is a strong influence on chromatin organization [24,33,35-39] and so it is necessary to control for expression level when examining additional potential influences. We first analyzed histone modifications previously associated with transcription elongation, that is, enriched within the bodies of expressed genes.

Trimethylation of the lysine 3 residue of histone H3 (H3K36me3) is catalyzed by Set2-type methyltransferases and is linked to transcription elongation [40-42]. As shown in Figure 1, CpG and non-CpG promoter genes with matched expression levels have very similar distributions of this modification, with a linear accumulation towards the 3' end of expressed genes, and levels that correlate with expression (Figure 1b). Only at the first nucleosome downstream of the site of initiation do highly expressed non-CpG promoters show a slightly higher level of this histone modification. The similar accumulation of H3K36me3 towards the end of the gene suggests a similar transcription elongation-coupled mechanism of H3K36me3 deposition in both gene classes.

### Transcription elongation-associated histone modifications peak in the 5' end of genes with CpG island promoters

**Figure 2:**
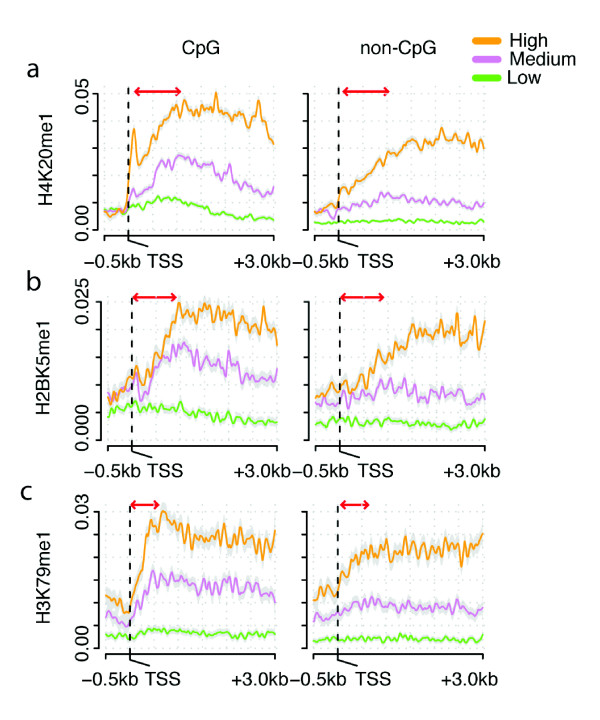
**Sharp increases in H4K20me1, H2BK5me1, and H3K79me1 modification levels in the bodies of CpG promoter genes**. **(a-c) **Average profiles of H4K20me1 (a), H2BK5me1 (b) and H3K79me1 (c) for expression-matched groups of CpG and non-CpG promoter genes (color-coded as in Figure 1a). Arrows indicate modification peaks unique to CpG promoter genes. TSS, transcription start site.

In contrast to H3K36me3, however, our analysis revealed that other histone modifications enriched within the bodies of active genes have different distributions in CpG and non-CpG promoter genes. The modification H4K20me1 is catalyzed by SETD8/Pr-Set7 methyltransferases and has been variously linked to gene silencing, gene activation, transcription elongation, and to the early exons of highly expressed genes [43-47]. In expressed genes with CpG promoters, H4K20me1 levels increase rapidly within gene bodies, reaching a peak on nucleosomes approximately 1 to 1.5 kb after the start site (Figure 2a). In contrast, in non-CpG promoter genes, H4K20me1 levels are low on promoter-proximal nucleosomes, increasing only gradually to a plateau approximately 2 to 2.5 kb after the start site (Figure 2a). A distinct peak is not observed, and non-CpG promoter genes with medium expression only have very low levels of H4K20me1 (Figure 2a).

Levels of the transcription elongation-linked modifications H2BK5me1 and H3K79me1/2/3 also differ between the two gene classes, and in a manner that is similar to H4K20me1. Modification levels peak on nucleosomes 750 bp to 1 kb downstream of CpG promoter genes (Figure 2b, c). In contrast, they show only weak monotonic enrichment in the bodies of genes without CpG promoters (Figure 2b, c; Additional file 3).

The exon density (Additional file 4), nucleosome density (see below) and DNA methylation levels (Additional file 5) downstream of highly expressed CpG and non-CpG promoters are very similar, and so cannot account for these differences in the deposition of transcription elongation marks (Additional file 3). Also, the differences in the chromatin profiles remain largely unchanged when a different CpG island definition is used (Additional file 6 using CpG islands defined by Hackenberg *et al. *[48] and Additional file 7 using the promoter classification of Weber *et al. *[25]), when we remove all genes with more than one transcription start site (Additional file 8), or even when we use an independent set of chromatin data from IMR90 cells (Additional file 9). Rather, genome-wide analysis suggests differences in the recruitment or activation of histone modifying enzymes during the early stages of transcription elongation in these two gene classes.

### Transcription-coupled shifts of histone modification peaks in the 5' end of CpG promoter genes

**Figure 3:**
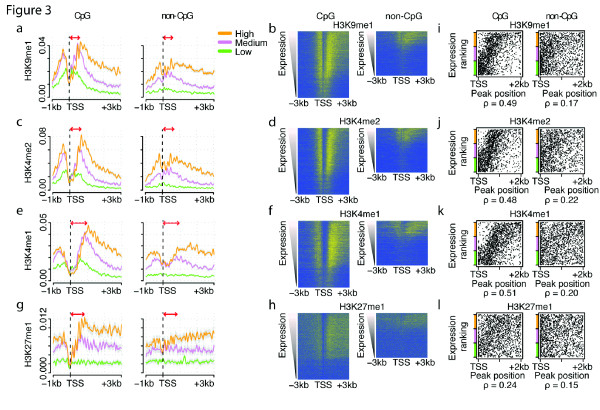
**Transcription-coupled shifts of histone modification peaks enriched in the 5' end of CpG promoter genes**. **(a-h) **Average profiles of expression-matched CpG and non-CpG genes (a, c, e, g) and heat maps of all genes (b, d, f, h) for H3K9me1 (a, b), H3K4me2 (c, d), H3K4me1 (e,f) and H3K27me1 (g, h). In the heatmaps, each row represents one gene and each column a single nucleotide position. **(i-l) **Plots of the correlation between expression level and peak modification position for H3K9me1 (i), H3K4me2 (j), H3K4me1 (k), H3K27me1 (l) at CpG and non-CpG promoter genes. The x-axis indicates the first position with the highest histone modification level within 2 kb of the gene transcription start site (TSS). The y-axis indicates the expression level ranking of the gene. For a direct comparison, only the expression-matched subsets of genes with low, medium and high expression are plotted here. The spearman rank correlation values are shown below the scatter plots (all *P*-values < 10^-8^).

A second class of histone modifications revealed by our analysis is a set of transcription-associated histone methylations that are enriched in genes with CpG island promoters, but for which the location of the most modified nucleosomes differs depending upon the expression level (Figure 3). As for the previously described modifications, the levels of the methylations H3K9me1, H3K4me2, H3K4me1 and H3K27me1 increase with expression. However, in genes with higher expression, maximum modification levels are found on nucleosomes located further into the body of a gene (Figure 3a-h). Indeed, the +1 nucleosomes of highly expressed genes have lower levels of these histone methylations than the +1 nucleosomes of lowly expressed genes (Figure 3i-l). To our knowledge, this shifting of histone methylation peaks into gene bodies has not been previously noted.

### Monomethylation of H3K36 is enriched in the bodies of genes with CpG island promoters

**Figure 4:**
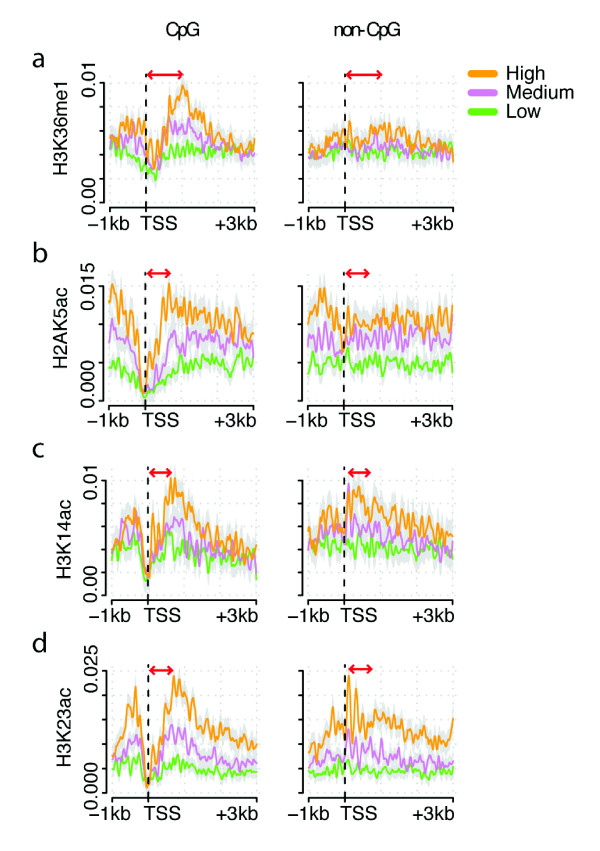
**Some transcription-associated histone modifications are depleted in the 5' ends of CpG promoter genes**. **(a-d) **Average profiles of expression-matched CpG and non-CpG genes for H3K36me1 (a), H2AK5ac (b), H3K14ac (c), and H3K23ac (d). Arrows indicate modification peaks unique to CpG promoter genes. TSS, transcription start site.

In contrast to H3K36me3, which is located towards the 3' end of active gene bodies, monomethylation of histone H3 lysine 36 (H3K36me1) is detected in the 5' end of highly expressed genes [46]. Surprisingly, our analysis suggests that H3K36 monomethylation is only enriched in the bodies of active genes that have CpG islands in their promoters (Figure 4a). Enrichment is detected on the fourth to eighth nucleosomes downstream of the start site, and then quickly returns to background levels within 2 kb (Figure 4a). In contrast, no enrichment is detected in highly expressed non-CpG promoter genes (Figure 4a). This suggests that the deposition of this modification may depend on the presence of a CpG island.

### Some transcription-associated histone modifications are depleted in the 5' ends of CpG promoter genes

In contrast to the histone modifications described above that are shifted closer to the transcription start site in CpG promoter genes (H4K20me1, H2BK5me1, H3K79me1/2/3), show stronger peaks that shift in location in CpG promoter genes (H3K9me1, H3K4me1, H3K4me2, H3K27me1), or are only enriched in CpG promoter genes (H3K36me1), a fourth set of modifications consists of transcription-associated modifications that are actually depleted in the 5' end of CpG island promoter genes. In highly expressed genes with CpG promoters, the modifications H2AK5ac, H3K14ac and H3K23ac peak approximately 750 bp after the initiation site (Figure 4b-d). In contrast, in non-CpG genes, these modifications either peak immediately after the start site (Figure 4c, d) or do not show a clear peak (Figure 4b). Thus, some transcription activation-associated histone modifications are depleted proximal to the start site of CpG promoter genes.

### A characteristic series of histone modification peaks means that the relative positions of histone modifications differ between genes with and without CpG island promoters

In expressed genes with CpG islands in their promoters, there is therefore a series of characteristic transitions in transcription-associated histone modifications in the region between approximately 0.5 and 2 kb after the initiation site. In contrast, in non-CpG genes, the same modifications gradually increase more distally within the gene body (H4K20me1, H2BK5me1, H3K79me1/2/3), show weaker or no enrichment within the gene body (H3K9me1, H3K4me1, H3K4me2, H3K27me1, H3K36me1), or are enriched from immediately after the initiation site (H2AK5ac, H3K14ac, H3K23ac). Thus, the identity, positioning, shape and relative ordering of transcription-associated histone modifications all differ between genes with and without CpG island promoters (Figures 2 to 4).

### Intrinsic binding preferences only predict nucleosome occupancy in non-expressed, non-CpG island promoters

**Figure 5:**
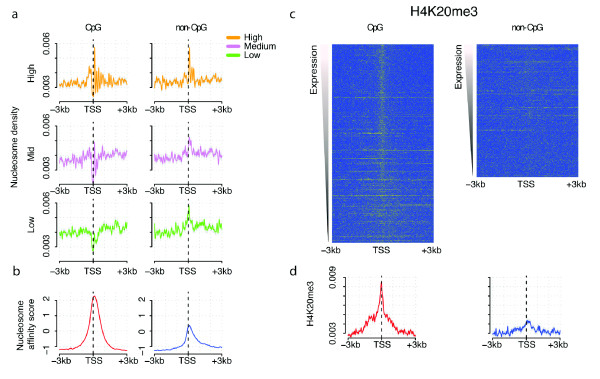
**CpG promoters have a constitutive nucleosome-depleted region and, when active, an unstable H4K20me3-modified nucleosome at the start site**. **(a, b) **Highly expressed CpG promoter and non-CpG promoter genes have an upstream nucleosome-depleted region and well-positioned nucleosomes within the gene body (a, top). However, CpG promoters also contain a nucleosome-depleted region when repressed (a, bottom left) whereas, in inactive non-CpG promoters (a, bottom right), nucleosome occupancy more closely resembles that predicted by the intrinsic binding preferences of nucleosomes for DNA (b). **(c, d) **In expressed CpG promoter genes the transcription initiation site within the nucleosome-depleted region (a) is precisely marked by H4K20me3.

We next compared overall levels of nucleosome occupancy within CpG and non-CpG promoter genes, as revealed by micrococcal nuclease digestion [49] (Figure 5). For highly expressed genes, patterns of occupancy are similar in the two gene classes: a characteristic nucleosome-depleted region is observed upstream of the transcription start site, and an array of well-positioned nucleosomes within the 5' end of the gene (Figure 5a). From an analysis of all genes it was previously proposed that nucleosome depletion upstream of transcription start sites is independent of gene expression level [49]. Our analysis showed that this is indeed the case for CpG promoters (Figure 5a), but for non-CpG promoters there is a clear association between a nucleosome-depleted region and mRNA expression levels (Figure 5a). That is, CpG promoters have a constitutive nucleosome-depleted region, but non-CpG promoters do not.

Moreover, comparing the nucleosome occupancy detected in human promoters to that predicted by the intrinsic binding preferences of nucleosomes for DNA [50] revealed that only in non-expressed, non-CpG promoters is nucleosome occupancy actually reflecting the intrinsic binding preferences of nucleosomes for DNA (Figure 5a, b). Thus, in contrast to the situation in transcriptionally quiescent sperm [51], in somatic cells influences beyond the affinity of the DNA for nucleosomes must be important for determining nucleosome occupancy in most active and repressed human promoters.

### Evidence for an unstable H4K20me3-modified nucleosome at the start site in active CpG island promoters

**Figure 6:**
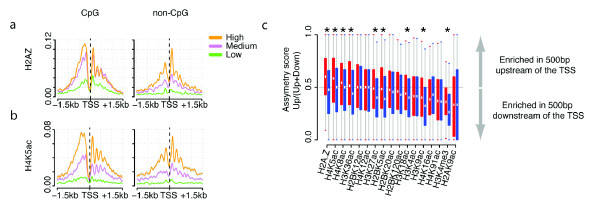
**Asymmetries in promoter proximal histone modifications around the transcription start site**. **(a,b) **H2A.Z **(a) and H4K5ac (b**) have higher modification levels upstream of the start site of CpG promoter genes. **(c) **The asymmetry of each promoter proximal modification is quantified for all highly expressed CpG (red) and non-CpG (blue) promoter genes. Here the number of modified histone reads between the transcription start site (TSS) and 500 bp upstream of the start site is divided by the number of reads between the start site and 500 bp downstream. Box plots indicate the range and 25, 50 and 75 percentile of the asymmetry score for the 500 highly expressed genes. Significant differences in the distributions between CpG and non-CpG promoter genes are indicated by an asterisk (Wilcoxon signed rank test, *P *< 0.05).

Consistent with the overall nucleosome depletion, most histone modifications are also depleted at the transcription start sites of CpG promoter genes and at the start sites of highly expressed non-CpG promoter genes (Figures 3, 4 and 6). However, our analysis revealed one notable exception to this: the modification H4K20me3 is detected precisely at the transcription start site in active CpG promoters (Figure 5c). This signal is weak but detected across many initiation sites (Figure 5d). With the current data, we cannot exclude the possibility that the signal is coming from a subset of cells within the population. Nevertheless, a similar peak is not seen for other modifications (Additional file 3). Previously, H4K20me3 has been linked to transcriptional pausing [52], to heterochromatin [53], and to the body of KRAB and zinc finger genes [39,46]. Our analysis suggests that the modification is also specifically enriched on an unstable nucleosome precisely positioned at the transcription start site in active CpG island promoters. To our knowledge, this is the first evidence for a specific histone post-translational modification precisely marking transcription start sites.

### Transcription-associated epigenetic asymmetries around start sites

Many epigenetic modifications, particularly histone acetylations, are enriched around the start sites of actively expressed genes (Additional file 3). One of these start site-proximal modifications, H3K4me3, was previously reported to show two distinct peaks in the forward and reverse directions, interpreted as corresponding to two divergent sites of RNA polymerase II initiation [16]. Our expression-matched gene sets show that these dual peaks are observed for many promoter-associated modifications, and for both CpG and non-CpG promoter genes (Figure 6a, b). Interestingly, however, the symmetry of these dual peaks about the start site can differ between CpG and non-CpG promoters. For example, the modifications H2A.Z and H4K5ac (and to a lesser extent H4K8ac, H3K36ac, H3K27ac, H2BK5ac, H3K18ac, H3K9ac and H3K4me3) actually have higher levels upstream of the start site (in the antisense orientation) than downstream (in the sense orientation) specifically in promoters with CpG islands (Figure 6). This difference is characteristic comparing across promoters (Figure 6) and again points to a different transcription-associated chromatin organization in genes with CpG island promoters.

### The chromosome organization factor CTCF may be bound immediately upstream of RNA polymerase II in most active CpG island promoters

**Figure 7:**
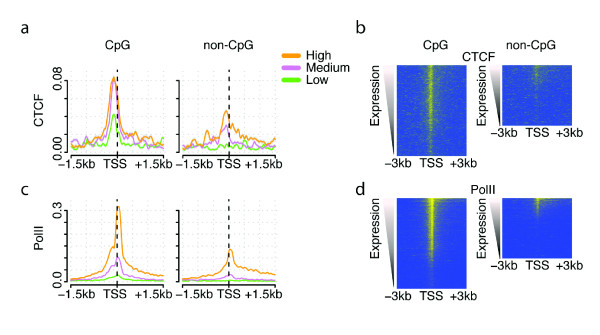
**CTCF is bound immediately upstream of RNA polymerase in active CpG island promoters**. **(a-d) **CTCF is bound immediately upstream of the initiation site, with similar recruitment in CpG genes with high and medium expression (a, b) and with high and low RNA polymerase II recruitment (c, d). TSS, transcription start site.

Finally, we examined the association of the chromosome organization CCTC-binding factor (CTCF) with CpG and non-CpG promoter genes, and how this association relates to gene expression. We chose to analyze CTCF because up to a quarter of all binding sites for CTCF have been reported to occur close to gene promoters [46,54]. Surprisingly, CTCF is detected at high levels immediately upstream of the start site in very many CpG island promoters (Figure 7a, b; Additional file 10), and at similar levels in genes with both medium and high expression (and medium and high levels of RNA polymerase II; Figure 7c, d). This suggests the intriguing possibility that CTCF binding might be part of the general architecture of an active CpG island promoter (see Discussion).

## Discussion

**Figure 8:**
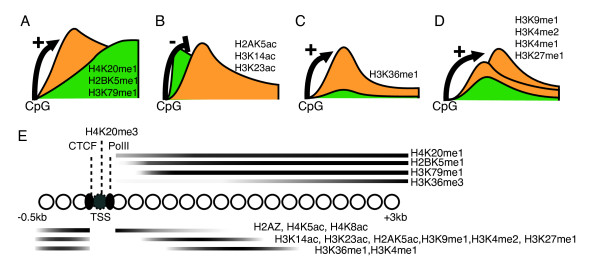
**Human genes with CpG promoters show distinct transcription-coupled changes in chromatin**. **(a) **Multiple transcription elongation-associated histone modifications are detected on nucleosomes closer to the transcription start site (TSS) in genes with CpG islands in their promoters (orange) compared to other genes (green). **(b) **In contrast, other transcription-associated histone modifications are excluded from a promoter proximal region. **(c) **H3K36me1 is only detected in the bodies of genes with CpG promoters. **(d) **Additional transcription-associated modifications are enriched in CpG promoter genes and shift in location away from the start as expression levels increase. **(e) **Taken together, this means that genes with CpG promoters show a characteristic series of modified nucleosomes upstream, downstream, and at the transcription start site.

We have presented here evidence that, depending upon the type of promoter in which transcription initiates, human genes show two distinct patterns of transcription-coupled changes in chromatin organization. Transcription from both CpG and non-CpG promoters is associated with a set of histone-modification transitions around the start site and into the gene body, but the identity, shape and ordering of these modifications differs between the two gene classes. These differences are summarized in Figure 8.

In the repressed state, CpG promoters are distinguished by a nucleosome-depleted region. Upon activation, this nucleosome-depleted region shows evidence of containing a nucleosome specifically modified by H4K20me3 at the initiation site, and CTCF binds immediately upstream of RNA polymerase. This suggests that CTCF could be part of the basic architecture of CpG island promoters, perhaps contributing to their organization into 'active chromatin hubs' or 'transcription factories' [55,56].

Multiple transcription elongation-coupled modifications (H4K20me1, H2BK5me1, H3K79me1/2/3) occur on nucleosomes closer to the transcription start site in genes with CpG promoters, suggesting an earlier and sharper transition in transcription elongation complexes. In contrast, a later elongation complex transition, as reflected in the deposition of H3K36me3, appears to occur independently of promoter type. H3K36me1 appears specific to CpG promoter genes, and H3K9me1, H3K4me1, H3K4me2 and H3K27me1 show stronger transcription-associated peaks in the 5' ends of these genes. Uniquely, the same modifications also shift into the bodies of genes as expression levels increase: the most modified nucleosomes are different in genes with high and low expression levels. This might, perhaps, reflect incompatibility with other modifications detected in higher levels close to the start site in highly active genes. Finally, other transcription-coupled modifications (H2AK5ac, H3K14ac, H3K23ac) are depleted in the very 5' end of CpG promoter genes. It is possible that enzymes removing these modifications are directly or indirectly recruited to CpG islands, as has been shown for the CXXC domain-containing H3K36me2 demethylase KDM2A [57].

## Conclusions

Taken together, therefore, we propose that active CpG promoter genes have a characteristic 'step-like' series of transitions in the modifications that nucleosomes carry upstream, downstream, and at the transcription initiation site, extending about 2 kb into a gene (Figure 8). Several potential molecular mechanisms could contribute to this characteristic chromatin signature. Possibilities include the direct recruitment of histone-modifying enzymes to CpG islands, a difference in the composition of RNA polymerase complexes loading in CpG island promoters, or altered dynamics of polymerase, such as delayed release from the promoter and different elongation speeds. CpG promoters often transcribe non-coding RNAs, and it is possible that some chromatin differences relate to the recruitment of chromatin-modifying enzymes by these RNAs [58]. The interplay between different modifications is also likely to be important: both the sequential recruitment of 'reader' and 'writer' proteins away from the start site and the incompatibility between different modifications could contribute to the establishment of the characteristic nucleosome series. For example, increased acetylase recruitment at the start site may contribute to the 3' shift in histone methylations in highly expressed CpG promoter genes. In future work, biochemical studies will be required to investigate these possible molecular mechanisms. Together with previous work, however, our results suggest that the chromatin organization of a human gene reflects not just the level of expression, but also the type of promoter in which transcription initiates.

## Materials and methods

### Gene start site and CpG island annotations

Human protein-coding genes were retrieved from Ensembl release 54 [59]. For each gene we considered only the most 5' transcription start site and we removed genes less than 3 kb long to avoid the inclusion of non-genic regions downstream of a gene. To avoid analyzing upstream regions that overlap another gene, we also removed all genes that have a promoter within 500 bp of another gene. We retrieved CpG islands from the UCSC genome browser [5,60]. We considered a gene as having a CpG-island promoter when its first transcription start site overlaps a UCSC CpG island. Removing genes with more than one transcription start site did not change any of the observations (see Additional file 8). All chromatin profiles were repeated using the alternative CpG island definition from [48] (shown in Additional file 6). Further, all chromatin profiles were repeated using the promoter definition by [25] (shown in Additional file 7). Following the original definition, to annotate promoters as 'high CpG promoters' (HCPs) we scanned the region from -1,200 bp to +300 bp of the transcription start site for a 500-bp window with CpG observed over expected ratio of > 0.75 and GC content > 55. Promoters with all windows with CpG observed over expected ratio ≤ 0.48 were annotated as 'low CpG promoters' (LCPs). The rest of the promoters were annotated as 'intermediate CpG promoters' (ICPs).

### Gene expression data

We retrieved MAS5 normalized mRNA expression data for CD4+ T cells from Schones *et al. *[49] (Gene Expression Omnibus (GEO) accession GSE10437), mapping U133-PLUS-2 probes to genes using Ensembl. Probes matching multiple genes were discarded. In total, 16,781 protein-coding genes had annotated expression levels. Genes were ranked according to their expression level, using the (replicate-averaged) value of the most sensitive probe. We then split all genes into three equally sized groups of genes according to their expression level (low, middle and high expression). From these we randomly sampled 500 low, 500 intermediate and 500 high expression non-CpG promoter genes. For each non-CpG promoter gene we then identified a CpG promoter gene with a very similar expression level (absolute difference between log_e _expression of non-CpG promoter gene and log_e _expression of CpG promoter gene ≤ 0.1). These three expression-matched sets of CpG and non-CpG promoter genes were used to generate average chromatin profile plots. The entire sets of high, intermediate and low expression genes were used to generate chromatin profile heatmaps (the number of genes in each class are included in Additional file 1). The expression-matched sets of genes and their annotations are included in Additional file 2. A small number of genes with extreme expression levels for which we could not find a similarly expressed gene with a different promoter type were removed. We repeated the analysis in exactly the same way for IMR90 cells using microarray gene expression data from Kim *et al. *[36] (GEO accession GSE2672).

### Chromatin profiles

We retrieved the mapped sequenced reads of nucleosome fragments [49] and ChIP-Seq mapped read data for H2A.Z, 20 histone methylations [46] and 18 histone acetylations [38]. Similarly, we analyzed DNA methylation data from the same cell-type [61]. These datasets contained all reads that match the genome in a unique position with up to two mismatches. To minimize sequence amplification bias, we removed identical reads. We shifted the start position of the reads by 75 bp in the direction of sequencing (75 bp is approximately half of the length of the isolated DNA fragments), this way transforming the read start positions to nucleosome dyad positions. All datasets were rescaled to 10 million uniquely mapped nucleosome fragments. To generate the average chromatin profiles shown in the figures, we counted the number of dyads that fall at each position along the region surrounding the gene start site. Smoothed lines were generated based on the per-base-pair averaged position-shifted read count using the loess regression function in R (with 180 bp span) [62]. The predict.loess R function was used for the calculation of 95% confidence intervals. For the background subtracted chromatin profiles included in Additional file 3 we used a 75-bp window sliding by 1 bp and calculated the difference between the number of shifted reads from the histone modification (or H2A.Z) and the number of shifted reads from the nucleosome occupancy. At each position with respect to the transcription start site we then calculated the mean and the standard error of the background-subtracted values assuming a Normal distribution. Heatmaps were generated using Java Tree View 1.1.5r2 [63]. We repeated all chromatin profiles using data from a fetal lung fibroblast cell line (IMR90) generated by the NIH Roadmap Epigenomics Project [64,65]. We downloaded the mapped reads provided as BED files. Because these reads were mapped to human genome version hg19, we converted all gene promoters from hg18 to hg19 using the LiftOver tool. These profiles are shown in Additional file 9. The accession identifiers of the samples used for these profiles are included in the figure legend. Regions of statistically significant CTCF binding in CD4+ T cells (used in Additional file 10), based on the data from Barski *et al. *[46], were retrieved from Ensembl (regulatory build of Ensembl release 68). We defined distal CTCF binding sites as those not overlapping any annotated Ensembl gene. The coordinates of CTCF peaks were converted from human genome assembly hg19 to hg18 using the liftOver tool [60].

### Sequence properties

Intrinsic nucleosome binding preference calculations along the human genome were predicted by Kaplan *et al. *and downloaded from the authors' website [50]. The average nucleosome affinity model score around the transcription start site of each gene set is shown in Figure 5b. We also carried out the same analysis using the predicted probability that a nucleosome will be formed at each genomic position [50], which gave very similar results (data not shown). Similarly, as a control, we also calculated the G+C content profile at ± 3 kb around the gene start of each group (Additional file 11).

### Histone modification peak positions and asymmetry around the gene start

To compare the asymmetry of a histone modification around the start of CpG promoter and non-CpG promoter genes, for each highly expressed gene we calculated the number of position-shifted reads that map to the 500 bp upstream of the gene start and divided by the total number of position-shifted reads that map within 500 bp both upstream and downstream of the gene start. We then compared the distributions of these 'asymmetry scores' at highly expressed CpG and non-CpG promoter genes using the Wilcoxon rank sum test.

## Abbreviations

bp: base pair; GEO: Gene Expression Omnibus.

## Competing interests

The authors declare that they have no competing interests.

## Authors' contributions

TV performed all analyses. TV and BL designed analyses and wrote the manuscript. Both authors read and approved the manuscript for publication.

## Supplementary Material

Additional file 1 **Table with gene groups classified according to promoter type and gene expression level**. Excel file containing human genes classified according to their promoter type and their expression level in CD4+ T cells and IMR90 cells. Click here for file

Additional file 2 **Table with the expression-matched groups of CpG and non-CpG promoter genes**. Excel file containing the Ensembl identifiers of genes and the corresponding expression values used to generate all the average chromatin profile plots in the main figures. Click here for file

Additional file 3 **Average background subtracted chromatin profiles of all histone modifications and H2A.Z at expression-matched CpG and non-CpG promoter genes**. Grey shading indicates the 95% confidence interval of the calculated mean. Click here for file

Additional file 4 **Exon density profiles of the expression-matched CpG and non-CpG promoter genes**. Click here for file

Additional file 5 **DNA methylation profiles at expression-matched CpG and non-CpG promoter genes**. Click here for file

Additional file 6 **Chromatin profiles of genes annotated according to whether they overlap CpG islands defined by the CpGcluster program **[**48**]. Click here for file

Additional file 7 **Chromatin profiles of genes grouped into three promoter types: high CpG promoters, intermediate CpG promoters and low CpG promoters**. For this supplementary figure, we annotated genes based on their promoter type using the definition of Weber *et al. *[25]. We sampled 300 genes from each expression level and each promoter type to generate the average chromatin profile plots. Click here for file

Additional file 8 **Chromatin profiles of genes that contain a single transcription start site**. For this supplementary figure, we removed all genes with more than one transcription start site. We then split the genes into the three expression levels and sampled 300 genes from each expression level and each promoter type to generate the average chromatin profile plots. Promoters are annotated according to UCSC downloaded CpG islands [5]. Click here for file

Additional file 9 **Chromatin profiles of expression-matched CpG and non-CpG promoter genes in IMR90 cells**. Note that the CD4+ T-cell chromatin profiles shown in the main paper and the IMR90 chromatin datasets shown here were generated with different chromatin immunoprecipitation protocols. In the case of CD4+ T cells, nucleosomes were isolated by micrococcal nuclease digestion before immunoprecipitation. In the case of IMR90 cells, chromatin was sonicated before immunoprecipitation. These experimental differences may account for some of the differences in the CD4+ T-cell chromatin profiles and the IMR90 chromatin profiles. The GEO file accession numbers for the datasets used here are GSE2672 (a), GSM521890 (b), GSM521915 (c), GSM521904 (d), GSM752986 (e), GSM521899 (f), GSM521895 (g), GSM521866 (h), GSM521881 (i), GSM521885 (j), GSM469975 (k). Promoters are annotated according to UCSC downloaded CpG islands [5]. Click here for file

Additional file 10 **CTCF binds CpG promoters more frequently than non-CpG promoters**. **(a-f) **CpG promoters are more frequently bound by CTCF than non-CpG promoters. The enrichment of CTCF at CpG promoters is independent of the definition of overlap (a, c, e). All comparisons have a chi-squared test *P*-value < 2.2e^-16^. To control for the correlation between CTCF binding and expression level, we split the two groups of genes into ten equally sized expression bins and counted the fraction of promoters overlapping CTCF binding sites in each expression bin (b, d, f). Error bars represent 95% confidence intervals of binomial proportions. **(g-i) **CTCF binding sites that overlap CpG promoters have similar levels of CTCF as intergenic CTCF binding sites. Here we show CTCF levels at all CpG promoters (g), CTCF levels at an equal number of distal CTCF binding sites (h) and CTCF levels at all intergenic CTCF binding sites (i). The CTCF profiles shown in (g-i) are centered on the CTCF peak mid-point. Click here for file

Additional file 11 **GC content around CpG and non-CpG promoter genes**. Click here for file
