# Supplementary figures and images for: Human genes with CpG island promoters have a distinct transcription-associated chromatin organization
Source: Genome Biol. 2012 Nov 27;13(11):R110. doi: 10.1186/gb-2012-13-11-r110 (PMC3580500; doi:10.1186/gb-2012-13-11-r110)

Additional file 3

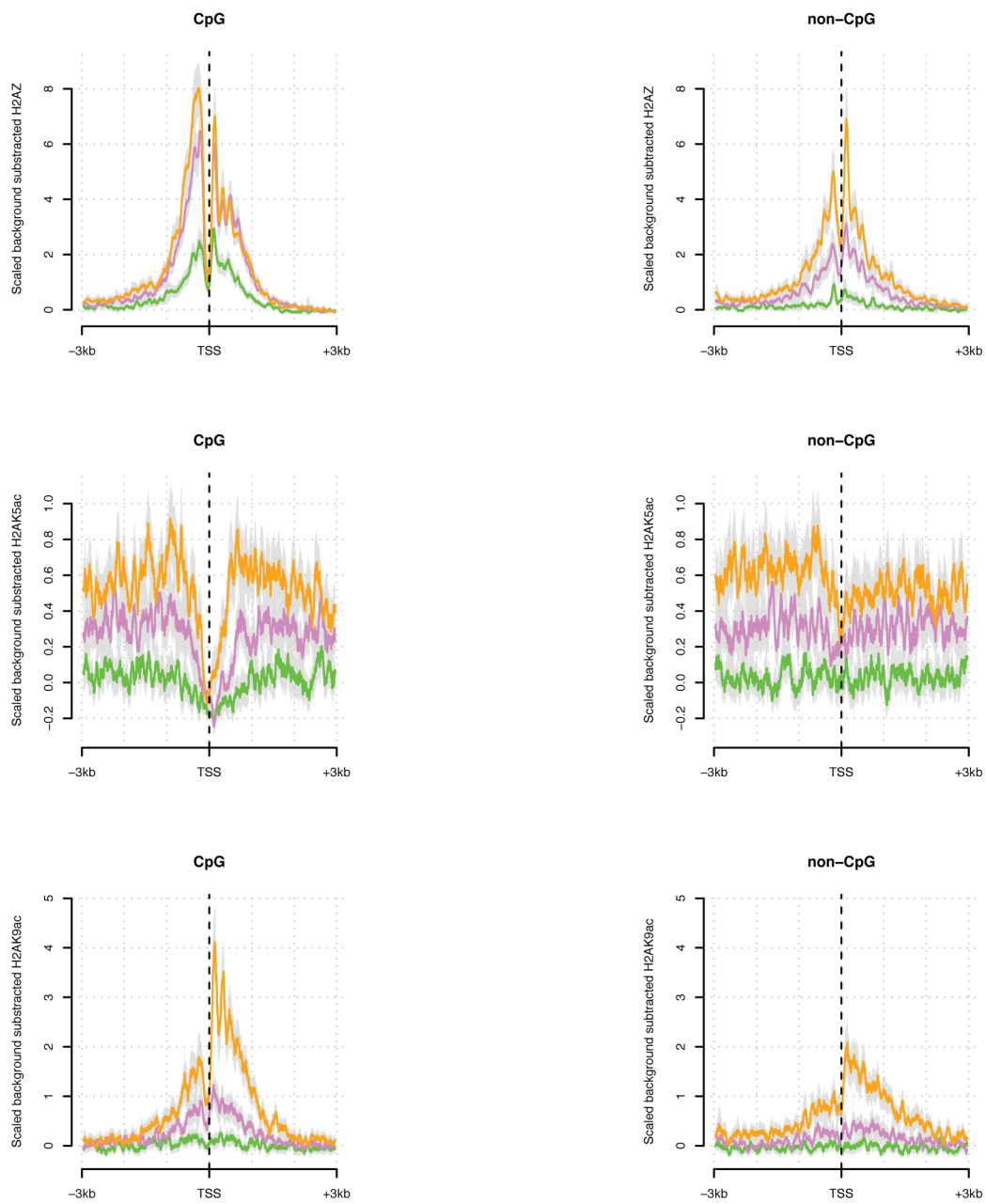

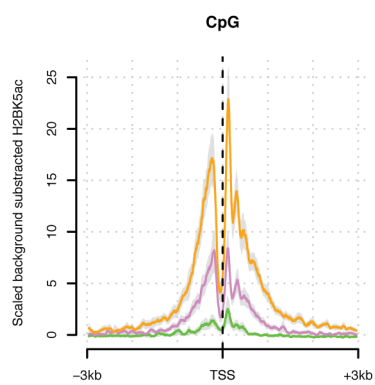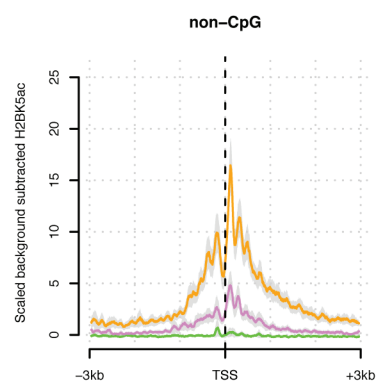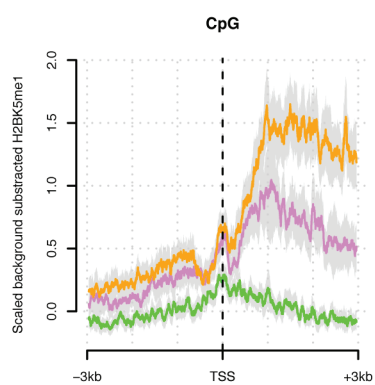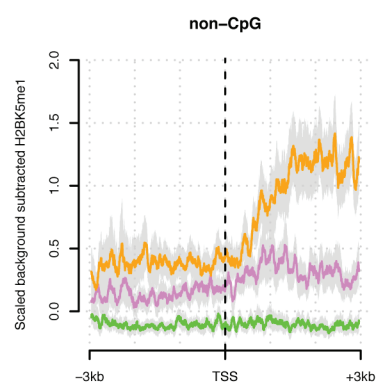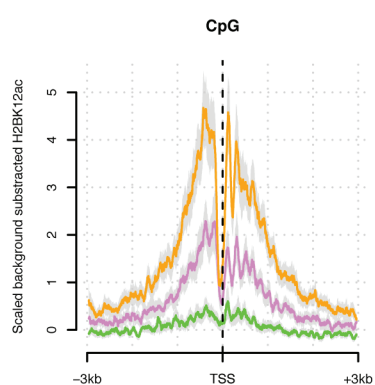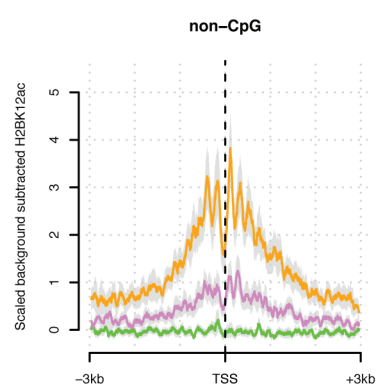

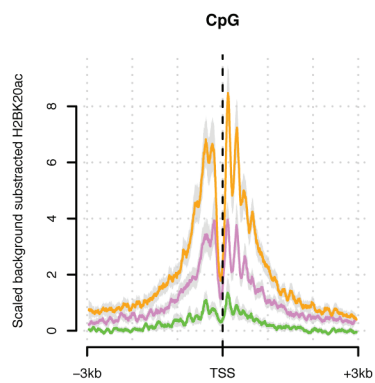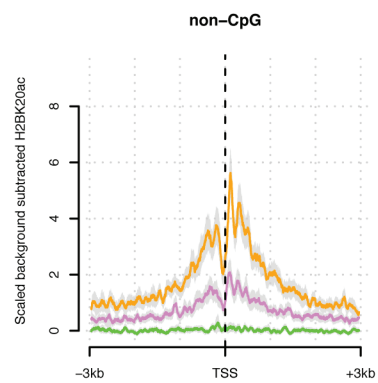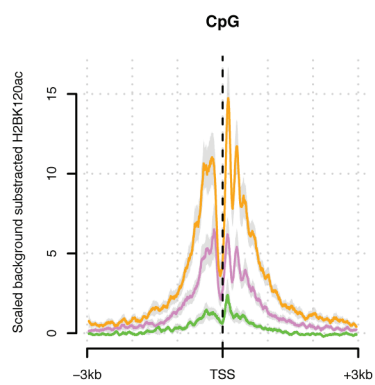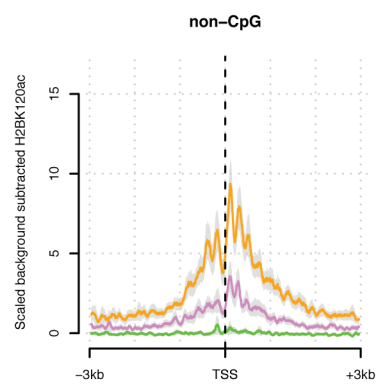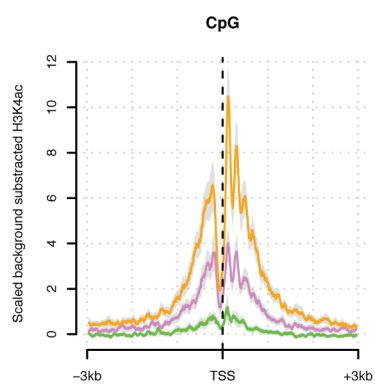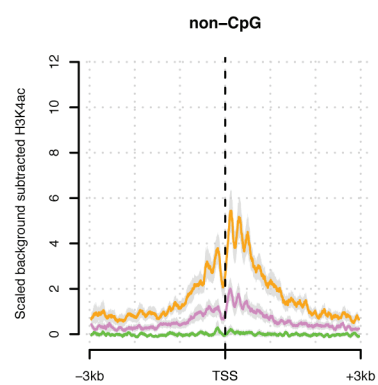

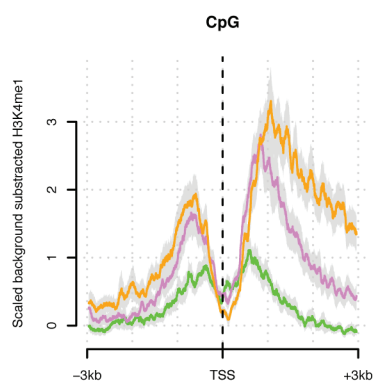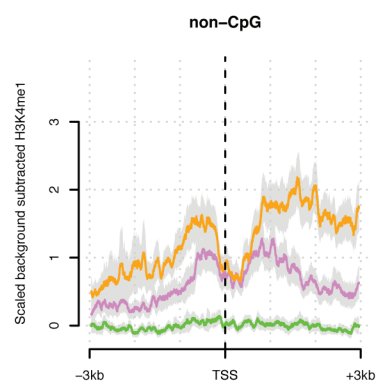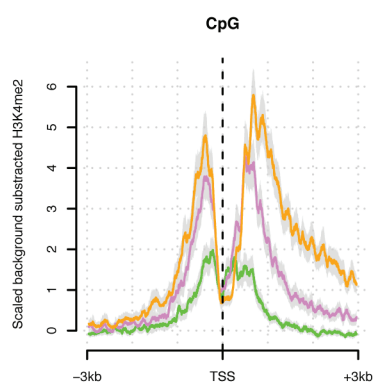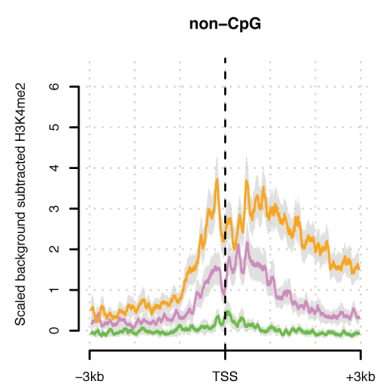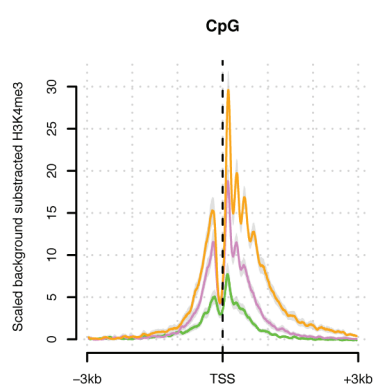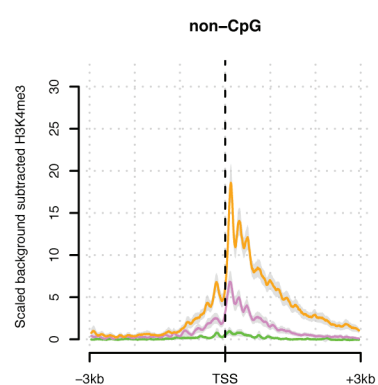

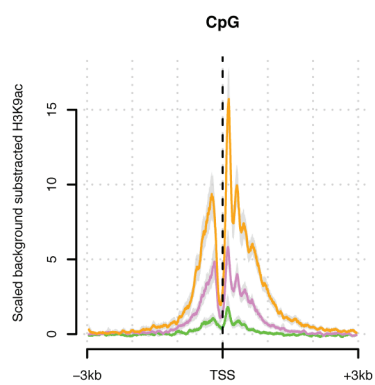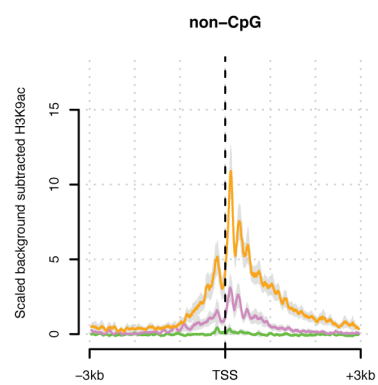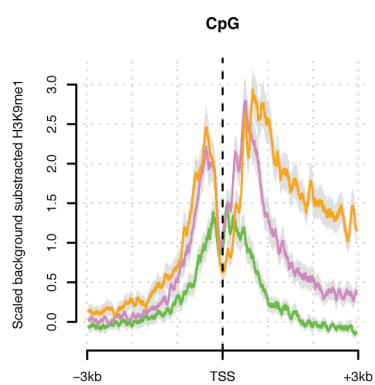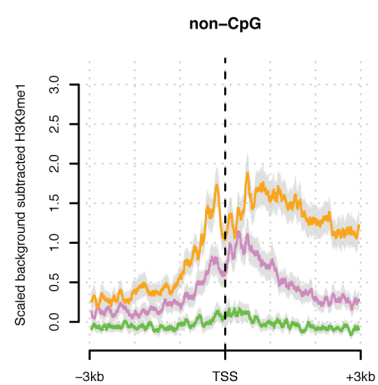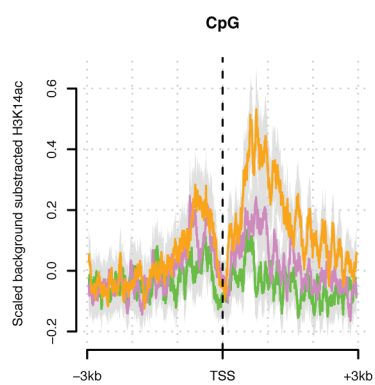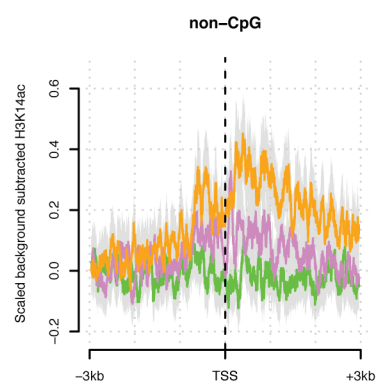

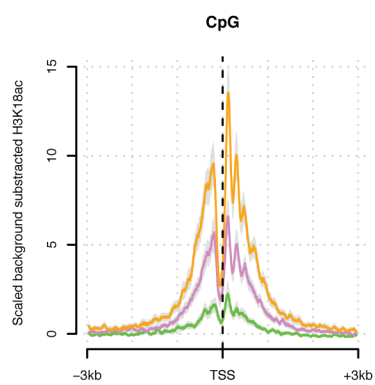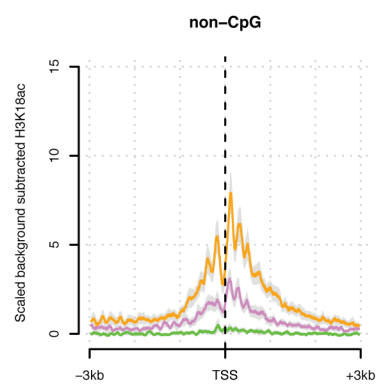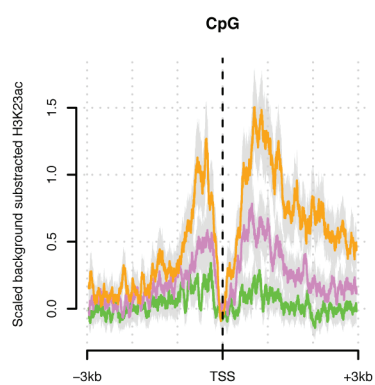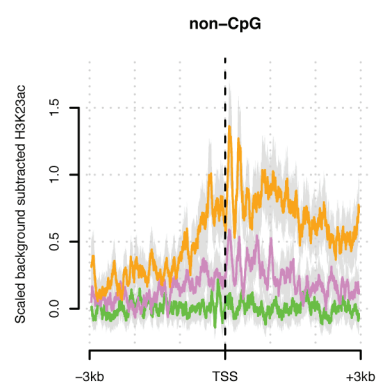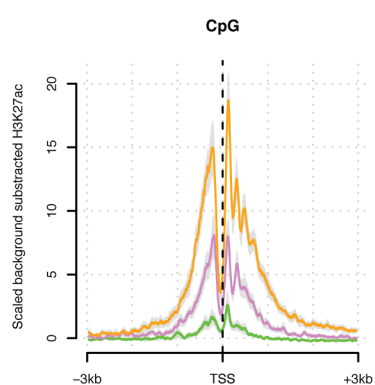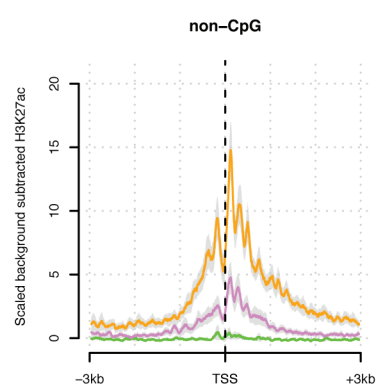

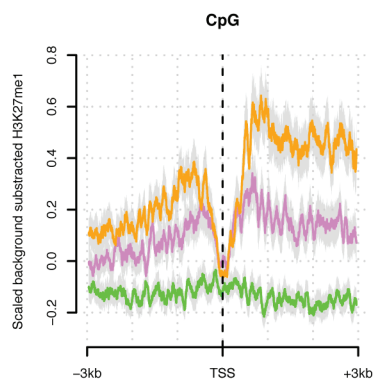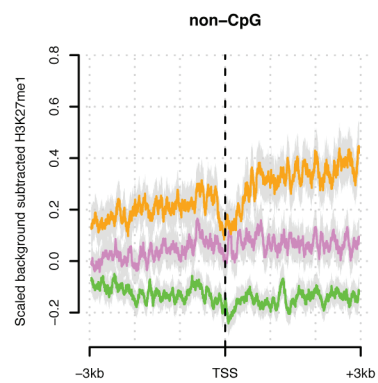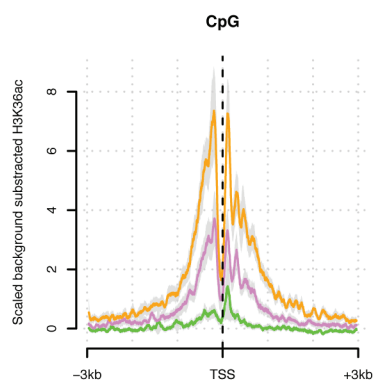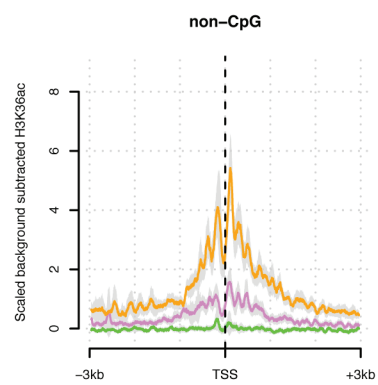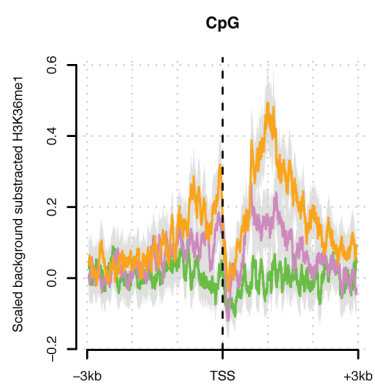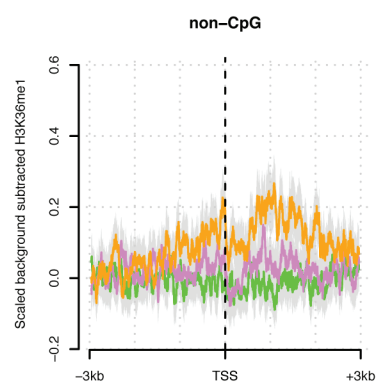

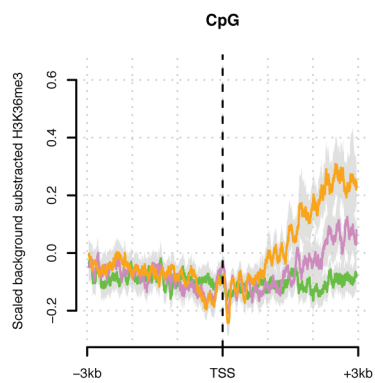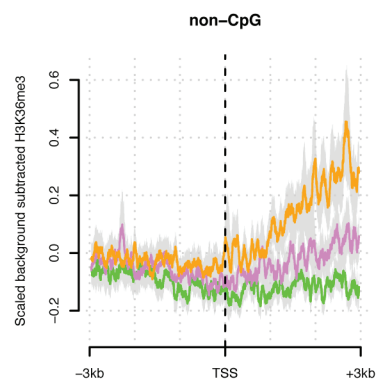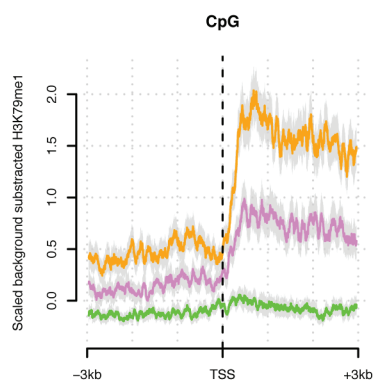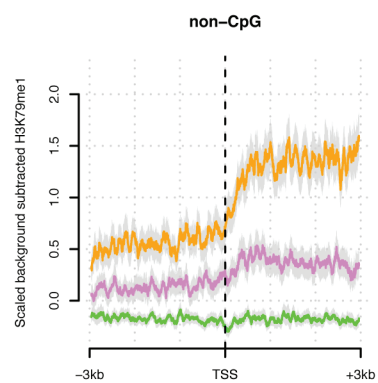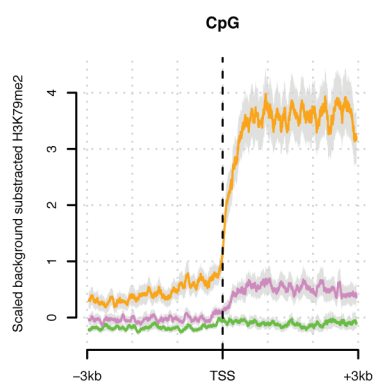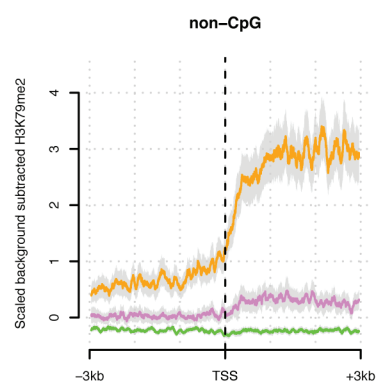

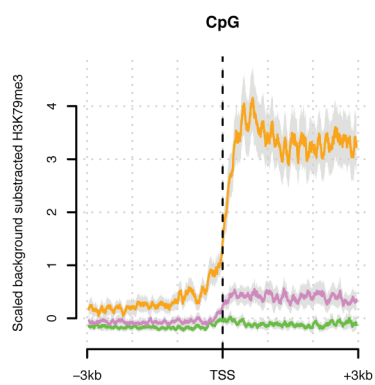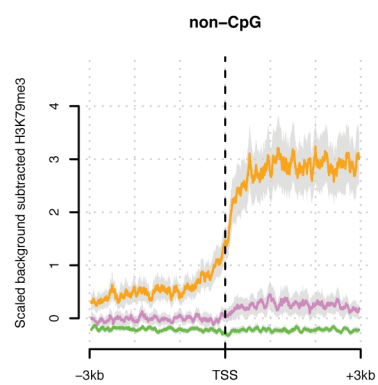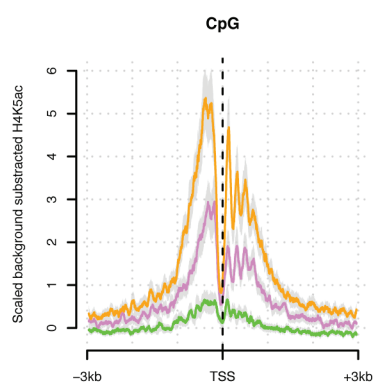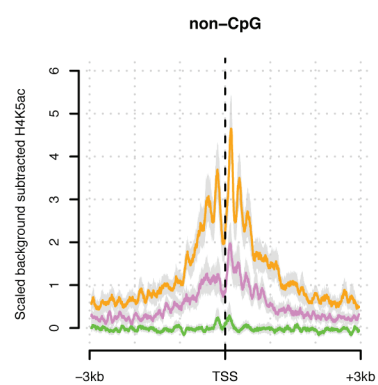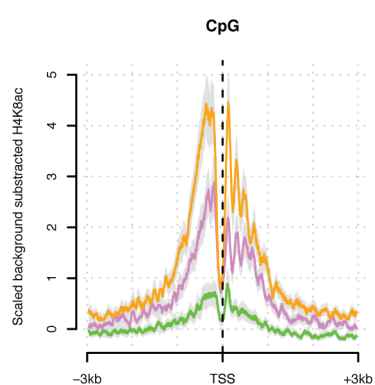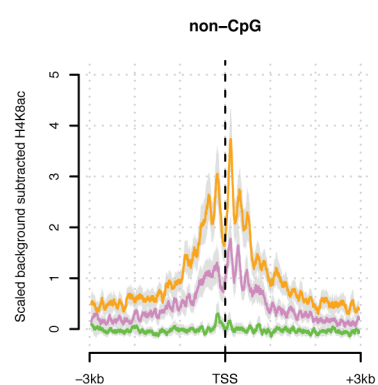

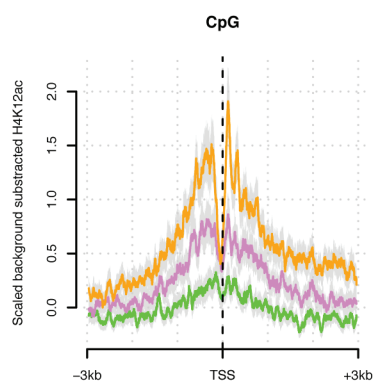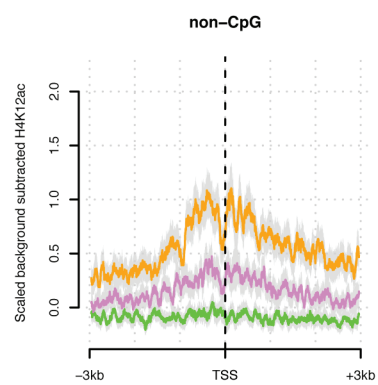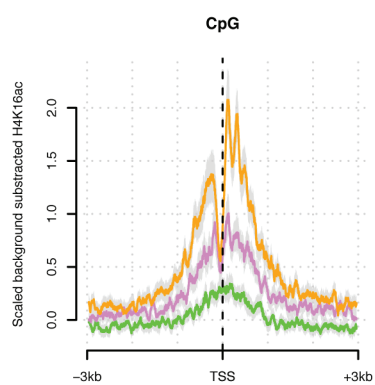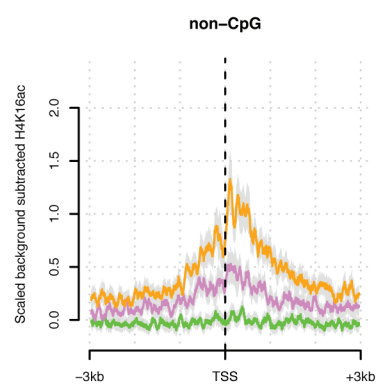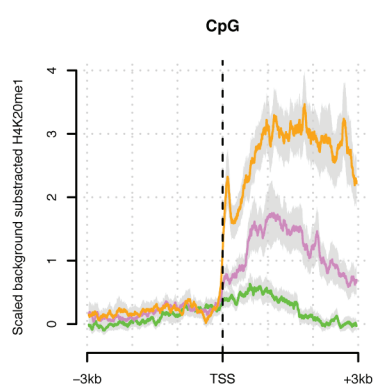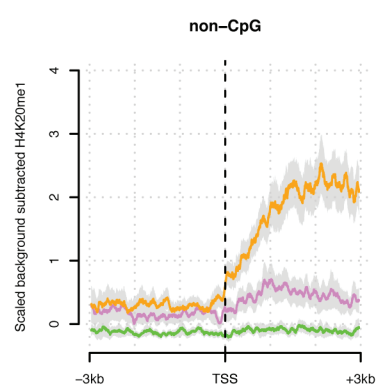

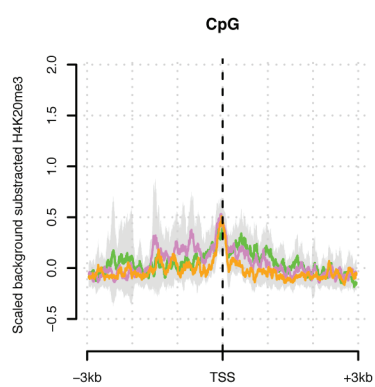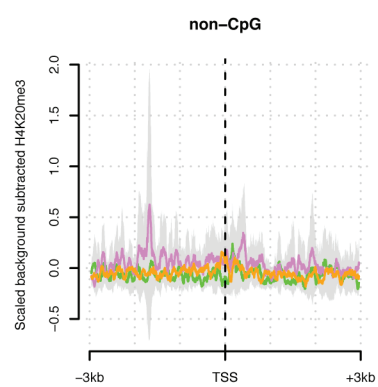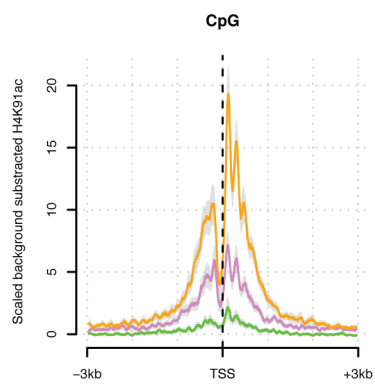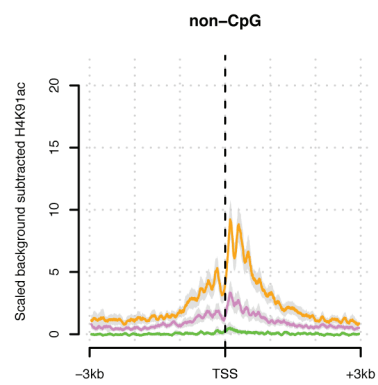

Supplement: Additional file 3 — Average background subtracted chromatin profiles of all histone modifications and H2A.Z at expression-matched CpG and non-CpG promoter genes. Grey shading indicates the 95% confidence interval of the calculated mean. [file gb-2012-13-11-r110-S3.PDF]

Additional file 4

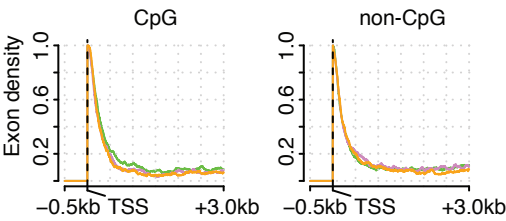

Supplement: Additional file 4 — Exon density profiles of the expression-matched CpG and non-CpG promoter genes. [file gb-2012-13-11-r110-S4.PDF]

Additional file 5

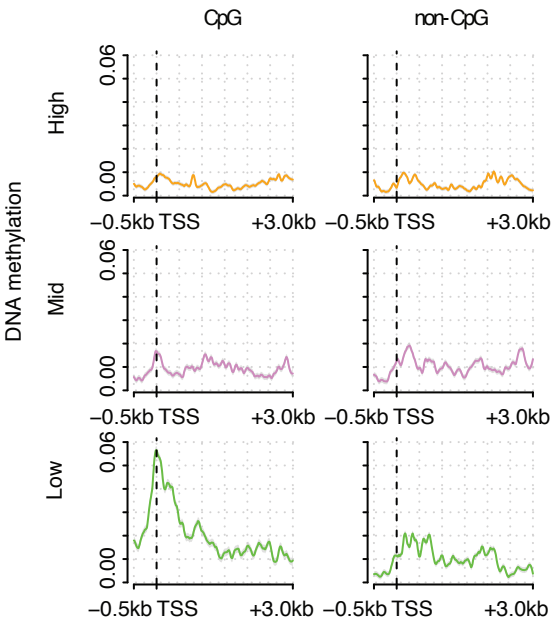

Supplement: Additional file 5 — DNA methylation profiles at expression-matched CpG and non-CpG promoter genes. [file gb-2012-13-11-r110-S5.PDF]

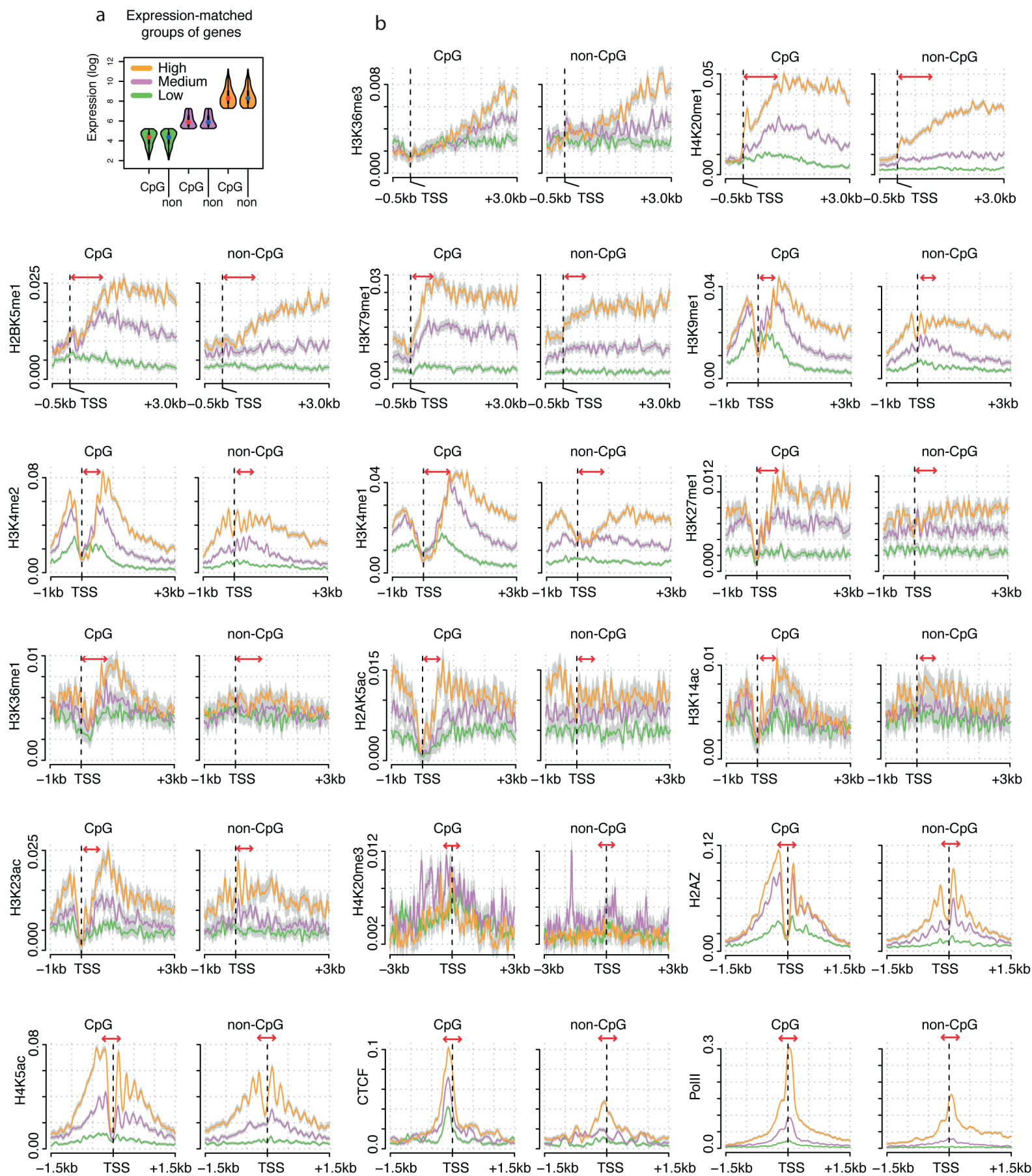

Supplement: Additional file 6 — Chromatin profiles of genes annotated according to whether they overlap CpG islands defined by the CpGcluster program [48]. [file gb-2012-13-11-r110-S6.PDF]

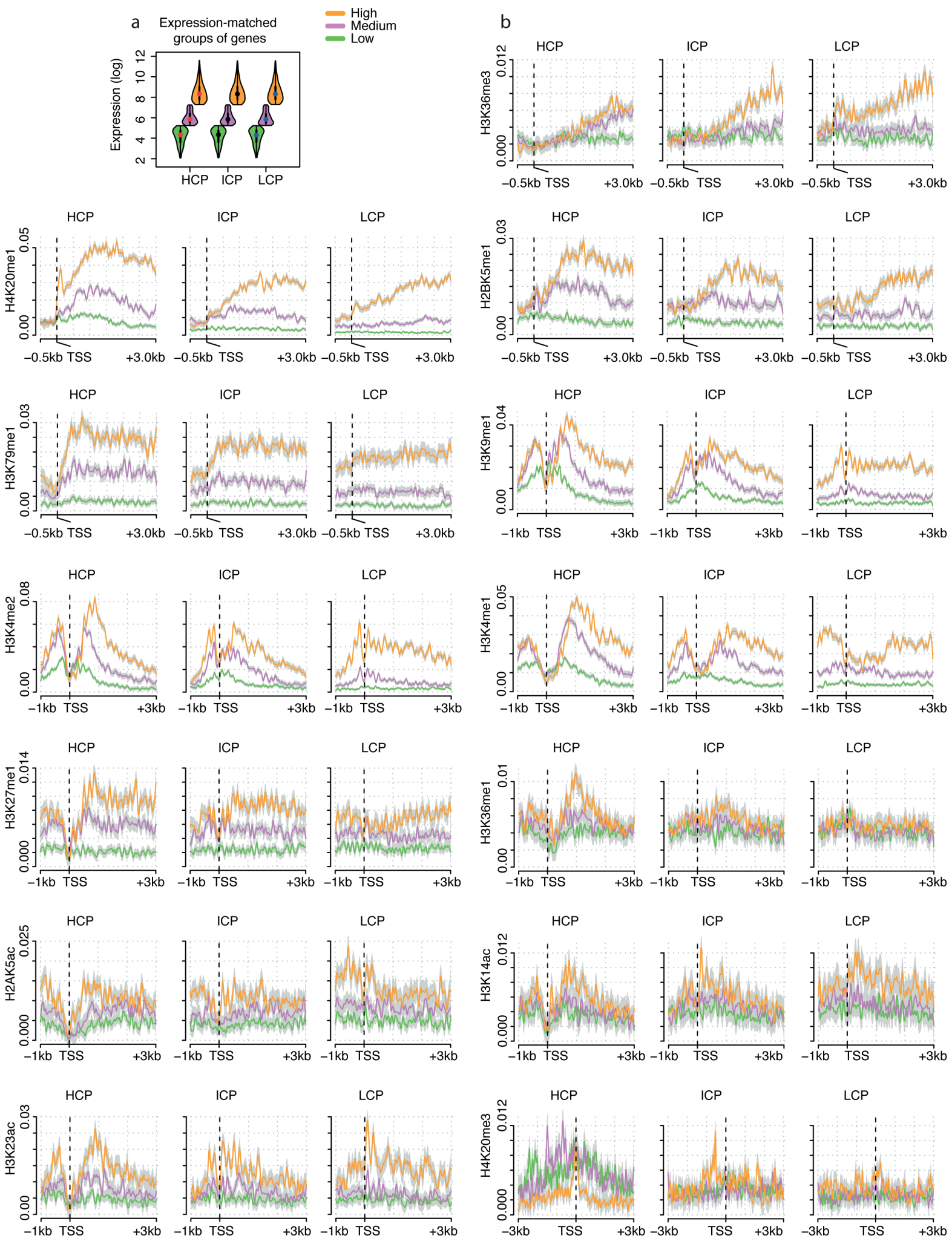

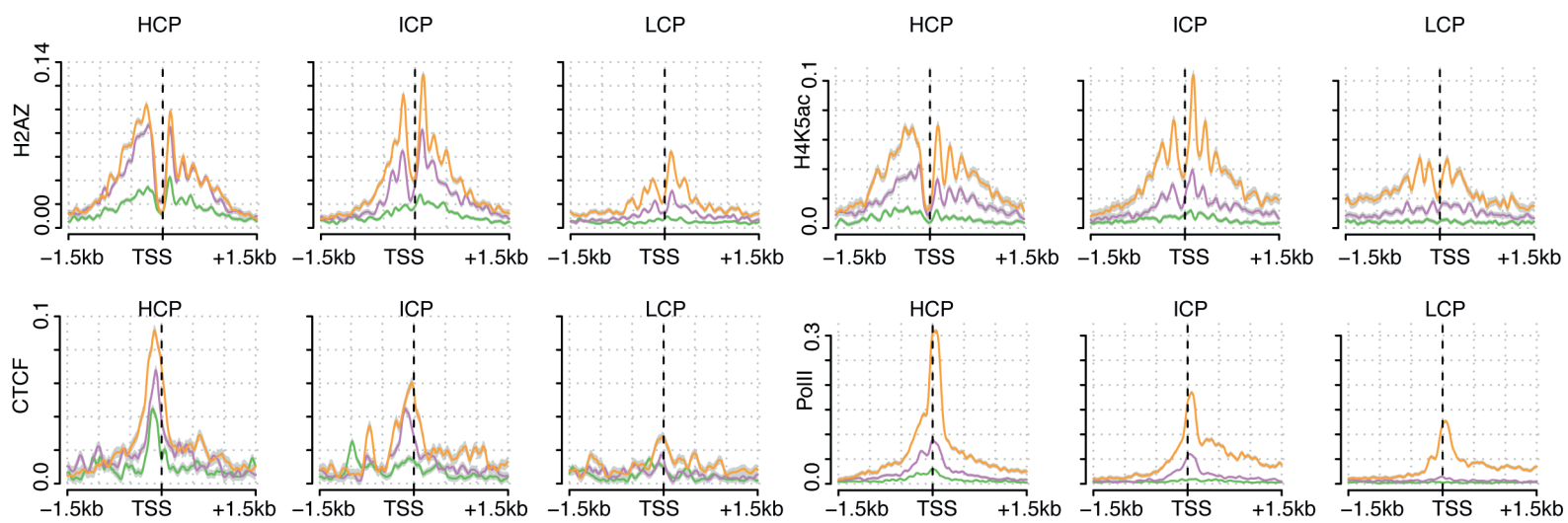

Supplement: Additional file 7 — Chromatin profiles of genes grouped into three promoter types: high CpG promoters, intermediate CpG promoters and low CpG promoters. For this supplementary figure, we annotated genes based on their promoter type using the definition of Weber et al. [25]. We sampled 300 genes from each expression level and each promoter type to generate the average chromatin profile plots. [file gb-2012-13-11-r110-S7.PDF]

a Expression-matched groups of genes

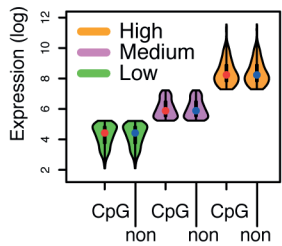

b

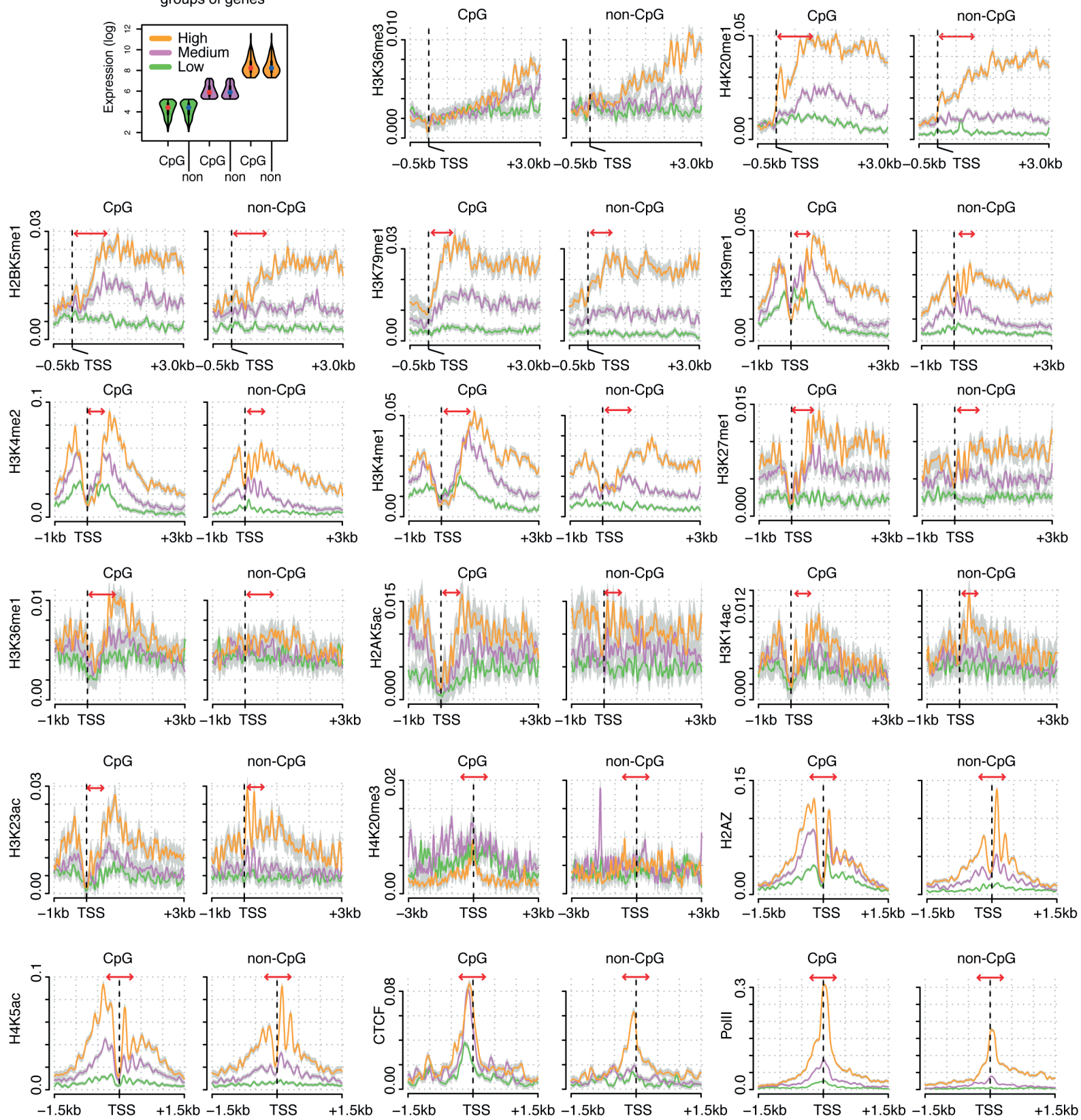

Supplement: Additional file 8 — Chromatin profiles of genes that contain a single transcription start site. For this supplementary figure, we removed all genes with more than one transcription start site. We then split the genes into the three expression levels and sampled 300 genes from each expression level and each promoter type to generate the average chromatin profile plots. Promoters are annotated according to UCSC downloaded CpG islands [5]. [file gb-2012-13-11-r110-S8.PDF]

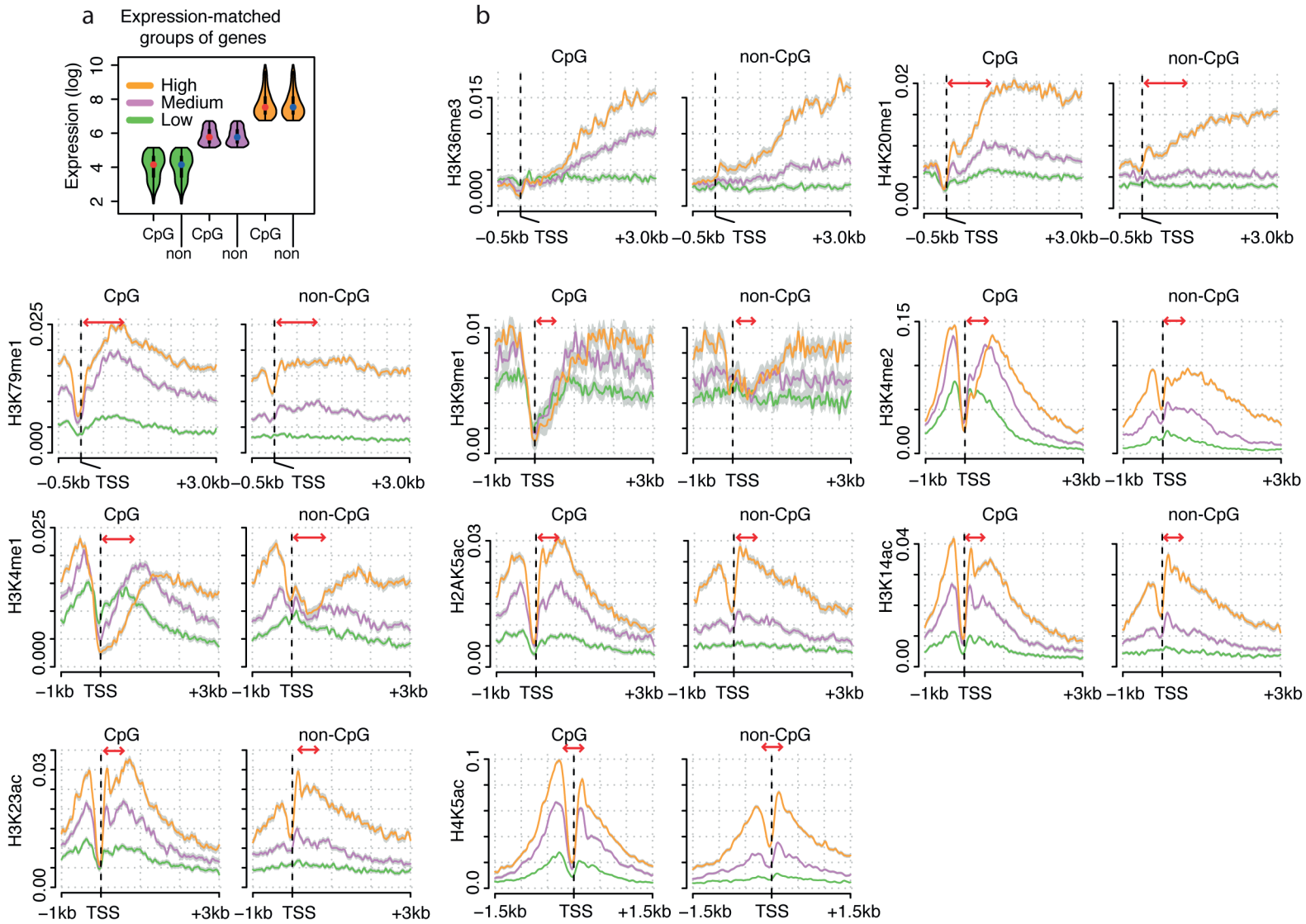

Supplement: Additional file 9 — Chromatin profiles of expression-matched CpG and non-CpG promoter genes in IMR90 cells. Note that the CD4+ T-cell chromatin profiles shown in the main paper and the IMR90 chromatin datasets shown here were generated with different chromatin immunoprecipitation protocols. In the case of CD4+ T cells, nucleosomes were isolated by micrococcal nuclease digestion before immunoprecipitation. In the case of IMR90 cells, chromatin was sonicated before immunoprecipitation. These experimental differences may account for some of the differences in the CD4+ T-cell chromatin profiles and the IMR90 chromatin profiles. The GEO file accession numbers for the datasets used here are GSE2672 (a), GSM521890 (b), GSM521915 (c), GSM521904 (d), GSM752986 (e), GSM521899 (f), GSM521895 (g), GSM521866 (h), GSM521881 (i), GSM521885 (j), GSM469975 (k). Promoters are annotated according to UCSC downloaded CpG islands [5]. [file gb-2012-13-11-r110-S9.PDF]

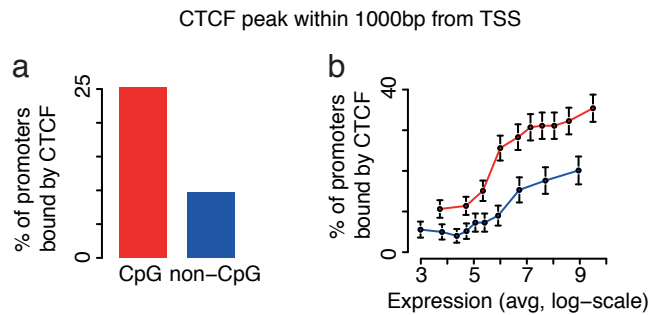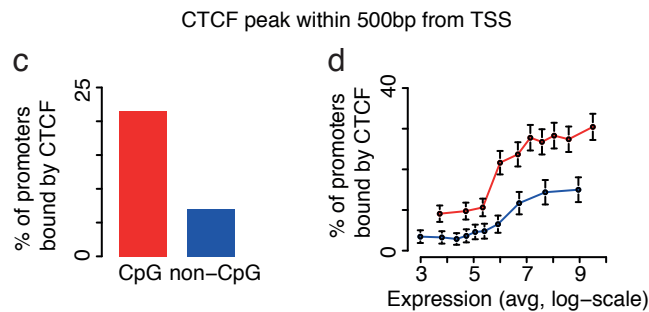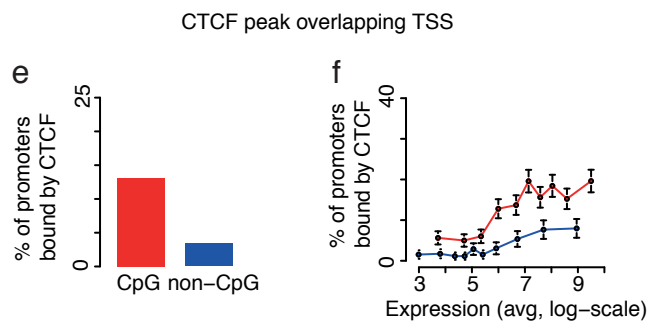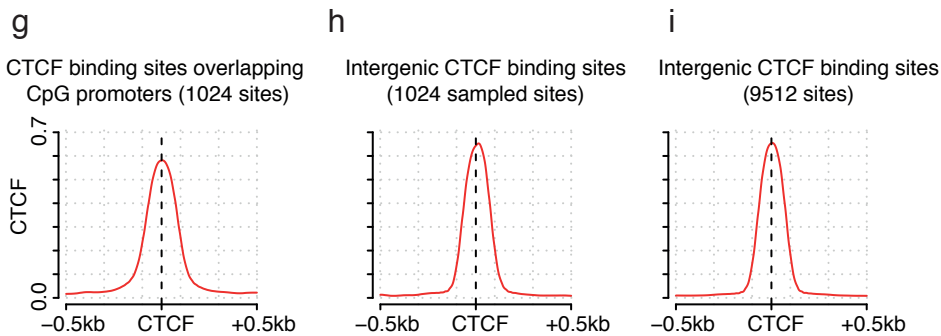

Supplement: Additional file 10 — CTCF binds CpG promoters more frequently than non-CpG promoters. (a-f) CpG promoters are more frequently bound by CTCF than non-CpG promoters. The enrichment of CTCF at CpG promoters is independent of the definition of overlap (a, c, e). All comparisons have a chi-squared test P-value < 2.2e-16. To control for the correlation between CTCF binding and expression level, we split the two groups of genes into ten equally sized expression bins and counted the fraction of promoters overlapping CTCF binding sites in each expression bin (b, d, f). Error bars represent 95% confidence intervals of binomial proportions. (g-i) CTCF binding sites that overlap CpG promoters have similar levels of CTCF as intergenic CTCF binding sites. Here we show CTCF levels at all CpG promoters (g), CTCF levels at an equal number of distal CTCF binding sites (h) and CTCF levels at all intergenic CTCF binding sites (i). The CTCF profiles shown in (g-i) are centered on the CTCF peak mid-point. [file gb-2012-13-11-r110-S10.PDF]

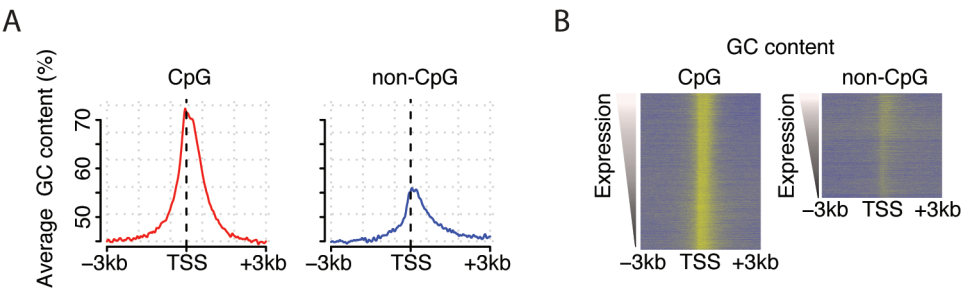

Supplement: Additional file 11 — GC content around CpG and non-CpG promoter genes. [file gb-2012-13-11-r110-S11.PDF]
